# Changes in Non-Structural Carbohydrates, Wood Properties and Essential Oil During Chemically-Induced Heartwood Formation in *Dalbergia odorifera*

**DOI:** 10.3389/fpls.2020.01161

**Published:** 2020-08-13

**Authors:** Zhiyi Cui, Xiaofei Li, Daping Xu, Zengjiang Yang

**Affiliations:** Research Institute of Tropical Forestry, Chinese Academy of Forestry, Guangzhou, China

**Keywords:** non-structural carbohydrates, essential oil, hydrogen peroxide, heartwood, *Dalbergia odorifera*

## Abstract

The highly valuable heartwood of *Dalbergia odorifera* T. Chen, known as *Jiang Xiang* in traditional Chinese medicine, is formed very slowly, and there is a need to better understand the process and promote heartwood formation. Chemical induction is considered to be one of the promising methods to induce heartwood formation. However, to date no method has been proved effective for *D. odorifera* as little is known about biochemical and physiological changes during heartwood development. Three potential heartwood induction substances *viz.* acetic acid, sodium chloride, and hydrogen peroxide solutions were injected into the trunk of *D. odorifera* to determine the effect on heartwood formation and physiological activity. Non-structural carbohydrates, lipids, wood properties, and essential oil were assessed in the post-treatment period. As also observed in the formation of natural heartwood, chemical-induced *Jiang Xiang* production was accompanied by sapwood dehydration, non-structural carbohydrates consumption, and synthesis of heartwood substances. As the heartwood substances accumulated, basic density and essential oil content increased gradually, thereby *Jiang Xiang* was finally produced. In this process, physiological parameters of discolored sapwood gradually evolved to resemble those of natural heartwood. Hydrogen peroxide-induced *Jiang Xiang* was closest to natural heartwood, and the essential oil components met the standards for high-quality *Jiang Xiang*, while the induction effects of acetic acid and sodium chloride were unsatisfactory. Thus, this study indicates that hydrogen peroxide has the potential to induce *Jiang Xiang* production in *Dalbergia odorifera*.

## Introduction

*Dalbergia odorifera* T. Chen (Leguminosae) is a medium-sized tree native to Hainan Island, southern China (Wariss et al., 2017) and has been widely cultivated in the tropical regions of Central and South America, Africa, and East and Southern Asia, especially in China (Ninh The, 2017). The heartwood of *D. odorifera* is not only one of the best rosewoods in the world, but is also a valuable traditional Chinese medicine known as *Jiang Xiang* (Yu et al., 2017; Liu et al., 2019). *Jiang Xiang* has been recognized by the Chinese Pharmacopeia for centuries to dissipate blood stasis, regulating *Qi*, stop bleeding, and relieve pain (Cheng et al., 1998; Wang et al., 2000; Sugiyama et al., 2002; Choi et al., 2009; Cui et al., 2017). Authoritative industry standards stipulate that high-quality *Jiang Xiang* has the relative amount of trans-Nerolidol 25–60%, (E)-beta-Farnesene 0–3%, and alpha-Bisabolol 0.1–6.0% in the essential oil (Li et al., 2016). The annual demand for raw *D. odorifera* heartwood is over 300 tonnes, and the annual production value exceeds 700 million USD. However, the heartwood of *D. odorifera* is formed very slowly once trees have reached about 6 years of age (Ma et al., 2017). Thus, there is a need to better understand and promote formation of *D. odorifera* heartwood (Cui et al., 2017).

Chemical injection appears to be the most promising technique for stimulating heartwood formation of trees as certain substances may act rapidly and are easy to apply in precise amounts. Potential heartwood induction chemicals include weak acids, inorganic salts and various plant growth regulators (ethrel, methyl jasmonate, salicylic acid), which have been applied to stimulate the production of agarwood, the heartwood of *Aquilaria sinensis* (Lour.) Gilg. (Liu et al., 2013; Van Thanh et al., 2015; Wang, 2016). In addition, chemical induction has also been used in *Acacia auriculiformis* A. Cunn. ex Benth. (Baqui et al., 1984), *Samanea saman* (Jacq.) Merr. (Patel and Bhat, 1984), *Quercus serrata* Thunb. (Moungsrimuangdee et al., 2011), *Santalum album* L. (Radomiljac, 1998; Liu, 2012), *D. odorifera* (Zhou et al., 2014; Wang et al., 2017), and some conifers (Stubbs et al., 1984; Martin et al., 2002). However, the chemical inducers in these studies are mostly plant growth regulators. It is still unclear whether weak acids and inorganic salts might induce the formation of *Jiang Xiang* in *D. odorifera*.

Mechanical wounding has been reported to induce the production of *Jiang Xiang* in *D. odorifera* (Meng et al., 2010). Moreover, hydrogen peroxide (H_2_O_2_) could be an important wound signal in *D. odorife*ra that may help induce vessel occlusions and production of *Jiang Xiang* (Cui et al., 2019). Experiments are required to determine the potential of chemicals such as hydrogen peroxide (H_2_O_2_), acetic acid (CH_3_COOH), and sodium chloride (NaCl) solutions.

The formation of heartwood is often accompanied by physiological processes such as xylem dehydration (Nakada, 2006; Kuroda et al., 2009), programmed cell death (Spicer, 2005; Nakaba et al., 2008; Nakaba et al., 2012), depletion of storage compounds (Magel et al., 1994; Piispanen and Saranpää, 2001; Nakaba et al., 2013), deposition of heartwood substances (Magel et al., 1991; Nakada and Fukatsu, 2012), and changes in cell wall structure (Nakada and Fukatsu, 2012; Song et al., 2014). The deposition of heartwood substances is the most important manifestation of heartwood formation due to its importance in natural durability; it is also important as heartwood extractives may be important pharmaceuticals. Heartwood substances are the products of secondary metabolism of trees, whose metabolized substrates are mainly non-structural carbohydrates (NSCs). NSCs in trees are the main photosynthetic storage compounds and transported inwards through ray parenchyma cells in the formation of secondary components in heartwood (Hillis, 1987). NSCs mainly include starch and soluble sugars (*e.g.* sucrose, fructose, glucose, arabinose, galactose, stachyose). The arabinose and galactose contents were reported to be related to the synthesis and hydrolysis of hemicellulose in the cell wall during the formation of heartwood (Saranpää and Höll, 1989; Fischer and Höll, 1992). In addition, the content of lipid in xylem has also proved to be involved in heartwood formation (Bergström, 2003).

In order to better understand heartwood formation induced by chemicals, this study was conducted to investigate: 1) whether H_2_O_2_, CH_3_COOH, and NaCl could induce the heartwood formation in *D. odorifera*; and 2) the physiological changes in xylem during chemical-induced *Jiang Xiang* production in *D. odorifera*.

## Materials and Methods

### Study Site

A 5-year-old *D. odorifera* plantation located in Xiashi Arboretum (22°60′ N, 106°53′ E), Pingxiang City, Guangxi Zhuang Autonomous Region (GZAR) was selected for this study. The site is characterized by the south subtropical monsoon climate with a mean annual temperature of 20.5–21.7°C, mean annual rainfall 1,200–1,500 mm yr^−1^, mean annual evaporation 1,261–1,388 mm yr^−1^, and mean annual relative humidity 80–84%. The soil is lateritic with an average depth of more than 1 m. The *D. odorifera* plantation was established in 2012 with 2 m × 2 m spacing. The status of stand growth in 2017 was as follows (mean ± standard deviation): tree height 5.03 ± 0.81 m, diameter at breast height (DBH) 6.54 ± 1.35 cm, and north–south crown diameter 3.21 ± 0.68 m.

### Experimental Design

Three chemicals used were: 0.1 mol L^−1^ hydrogen peroxide (H_2_O_2_), 1.0 mol L^−1^ (pH≈2.4) acetic acid (CH_3_COOH) and 1.0 mol L^−1^ sodium chloride (NaCl). Distilled water was injected as a control. Sixty trees of similar size, all without heartwood were selected for a single-tree plot experiment with 15 replicates for each treatment. A tiny drill was used to detect which trees have or don’t have heartwood. In May 2017, an injection hole of 1 cm diameter and 5 cm deep was drilled at a downward angle of 45° at 1.3 m from the ground on each tree. An amount of 50 ml solution was injected into each tree over 8 h period with a 5 ml syringe, and the hole was sealed with a cork after injection.

### Field Sampling and Measurement

The DBH, tree height, and north–south crown diameter of the sampled trees were measured before and at 6 months after commencement of treatment, and the growth increment was calculated.

**Figure 1:**
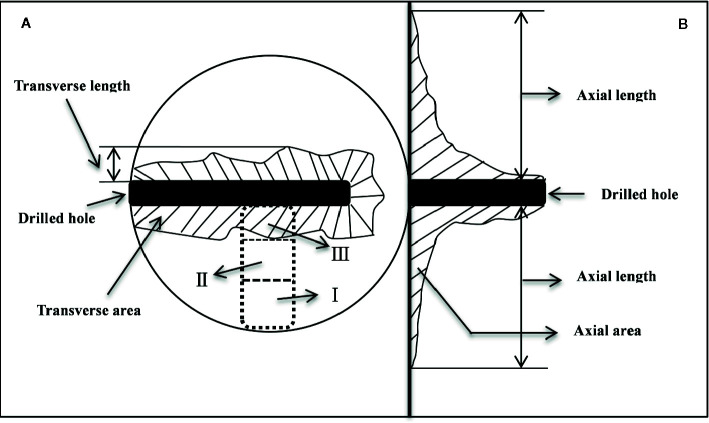
Schematic diagram of the discoloration range measurement and sampling positions. **(A, B)** indicate the transverse and axial anatomy, respectively. In the dotted box, the normal sapwood was divided into positions I and II on average, and the discolored part was defined as position III.

Five trees per treatment were harvested at one, three and six months after injection treatment. At the same time, three trees with heartwood were selected to collect natural heartwood. At each harvest, the trees were felled, and the boles were immediately dissected (**Figure 1**). Wood samples were sequentially collected from the cambium to the pith as shown in **Figure 1A**. In the dotted box, the normal sapwood was divided into positions I and II, and the discolored part was defined as position III. These samples were immediately deactivated in a 600-W microwave oven for 90 s (Hoch et al., 2002), dried at 65°C to constant weight, ground and passed through a 50 mesh sieve, and stored in a refrigerator at 4°C for determination of NSCs and lipids.

The length of discoloration in the transverse (**Figure 1A**) and axial directions (**Figure 1B**) was measured with a ruler, and the irregular area of the discoloration was determined by the grid-area method (Gao, 2015).

### Laboratory Determination

#### NSCs and Lipids

Arabinose and galactose contents were determined on an Agilent 1200 liquid chromatograph with the following parameters: SHISEIDO C18 column (4.6 × 250.0 mm, 5 μm); mobile phase: 0.1 mol L^−1^ pH 7.0 phosphate buffer solution, acetonitrile:water = 82:18 (v:v); flow rate 1.0 ml min^−1^; column temperature 25°C; injection volume 10 μl; wavelength 245 nm (after degraded by 4 mol L^−1^ trifluoroacetic acid and derivatives with 1-phenyl-3-methyl-5-pyrazolone). Glucose, fructose, sucrose, and stachyose were determined by high performance liquid chromatography (HPLC) with the following parameters: Shodex NH2P column (250.0 × 4.6 mm, 5 μm); mobile phase: acetonitrile:water = 75:25 (v:v); flow rate 1.0 ml min^−1^; injection volume 10 μl; column temperature 35°C; detector: differential refractive index (DRI), temperature: 35°C. The starch content was determined by an anthrone colorimetric method (Osaki et al., 1991). The lipid content was determined by Soxhlet extraction as described in detail by Ramluckan et al. (2014).

#### Relative Moisture Content and Basic Density

Fresh wood samples were weighed immediately three times, and basic density (bone-dry weight per unit of fresh volume) was determined using the water displacement method. Dry weight was obtained after drying at 105°C to constant weight, and relative moisture content was calculated (Searle and Owen, 2005).

#### Extraction and Component Analysis of Essential Oils

For each treatment, 5 g powdered wood samples were immersed in 50 ml petroleum ether and shaken for 24 h. After filtration and concentration (Concentrator 5301, Eppendorf, Germany), essential oils were obtained, and the oil content was calculated (Cui et al., 2019). The oils were kept at 4°C until analysis. The extractions were repeated three times and the oil content was reported as a percentage of the dry weight.

GC–MS analysis was performed by Agilent coupled with a 6890N-5975I system equipped with flame ionization detector (FID) and a DB-5MS (30 m × 0.25 mm, 0.25 μm film thickness). The temperature program included a starting temperature of 70°C which was then increased to 250°C at the rate of 8°C min^−1^, and this temperature held for 15 min. Detailed operating parameters are described by Cui et al. (2019). Qualitative identification of essential oil components was based on comparison of their retention times and mass spectra with the data in the Wiley and NIST electronic libraries as well as the authentic reference compounds that were reported in the published literature (Zhao et al., 2000). The relative amount of each component was calculated by comparing its average peak area to the total areas.

### Statistical Analysis

Data were subjected to one-way analysis of variance (ANOVA), and any significant differences among H_2_O_2_, CH_3_COOH, NaCl, and control treatments at one, three, and six months after treatment were evaluated using Duncan’s multiple range tests using the data processing software SPSS 17.0 (IBM, United States). Significant differences in non-structural carbohydrates and lipid contents in each part of the xylem among H_2_O_2_, CH_3_COOH, NaCl, and control treatments were evaluated by one-way ANOVA with asterisks, ^*^ *p* < 0.05, ^**^ *p* < 0.01. The plots for the graphs were generated in SigmaPlot 10.0 (Systat, United States).

## Results

### Effect of Chemical Induction on Tree Growth

**Figure 2:**
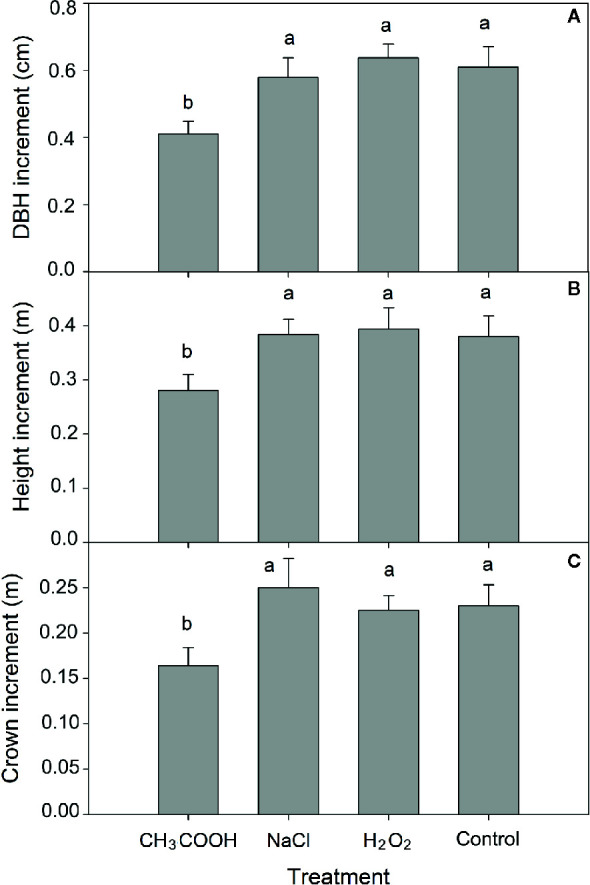
Effects of three chemicals on tree growth. **(A–C)** indicate the increment of DBH, tree height, and tree crown after 6 months of treatment, respectively. Similar letters indicate no significant difference at *p* > 0.05. Bar represents standard deviation.

After six months of treatment, significant differences in increment of tree height, DBH, and tree crown diameter were observed between chemical treatments (*p* < 0.01). Compared to control, the increment in height, DBH, and crown diameter of the CH_3_COOH treated trees was significantly decreased by 25.53, 34.92, and 32.78%, respectively (**Figure 2**). There were no significant differences between NaCl, H_2_O_2_, and control treatments. These results indicated that the CH_3_COOH treatment markedly inhibited tree growth, while the drill hole control, NaCl, and H_2_O_2_ treatments had little effect or no effect on tree growth.

### Effect of Chemical Induction on NSCs and Lipid in Xylem

**Figure 3:**
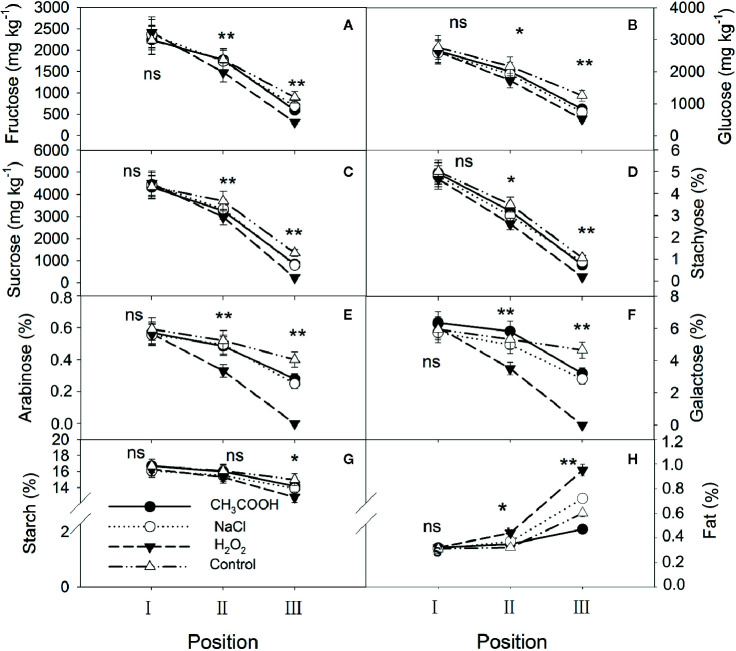
Non-structural carbohydrates and lipid contents in parts of the xylem after one month of treatment. **(A–H)** indicate fructose, glucose, sucrose, stachyose, arabinose, galactose, starch, and fat, respectively. Significance of statistical analysis (one-way ANOVA) shown with asterisks, ^*^ *p* < 0.05, ^**^ *p* < 0.01. “ns” indicates no significant difference at the 0.05 level. Bar represents standard deviation.

One month after injection, significant changes occurred in the starch, soluble sugars, and lipids in various positions of the xylem. For all treatments, NSCs gradually decreased while lipids increased from position I to position III (**Figure 3**). There were no significant differences in starch, soluble sugars, and lipids in position I between various treatments (*p* > 0.05). For position II, except for starch, significant differences in NSCs were observed between different treatments, with the H_2_O_2_ treatment being the lowest. The lipid content showed the opposite trend. Contents of starch, soluble sugars, and lipids in position III were all significantly different among the treatments, and their patterns were similar to those of position II. In addition, there were lower concentrations of stachyose, galactose, arabinose, and sucrose than other NSCs in position III, especially for the H_2_O_2_ treatment. Correspondingly, the lipid content in position III was higher in the H_2_O_2_ than in other treatments.

**Figure 4:**
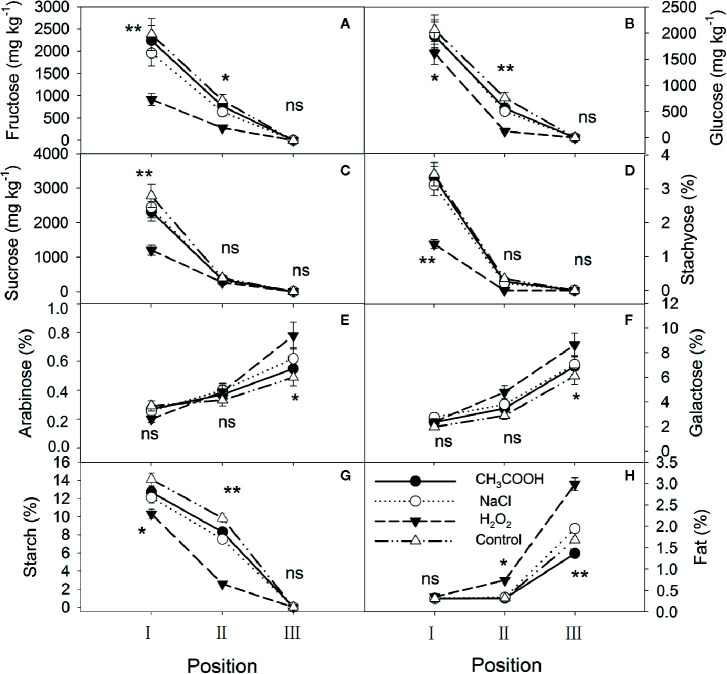
Non-structural carbohydrates and lipid contents in parts of the xylem after six months of treatment. **(A–H)** indicate fructose, glucose, sucrose, stachyose, arabinose, galactose, starch, and fat, respectively. Significance of statistical analysis (one-way ANOVA) shown with asterisks, ^*^ *p* < 0.05, ^**^ *p* < 0.01. “ns” indicates no significant difference at the 0.05 level. Bar represents standard deviation.

After six months of treatment, significant changes were observed in each treatment (**Figure 4**). Fructose, glucose, sucrose, stachyose, and starch in position I were significantly different among treatments, with the contents in the H_2_O_2_ treatment being the lowest, while arabinose, galactose, and fat were not significantly different. Except for sucrose and stachyose, the other carbohydrates in position II almost continued the patterns of the position I, and the lipid content was also significantly different between treatments. Furthermore, compared to one month after treatment, fructose, glucose, sucrose, stachyose, and starch were depleted in position III in all treatments, whereas the arabinose and galactose contents significantly increased (**Figures 4E, F** **)**. The lipid content in positions II and III increased significantly in all treatments over time. In particular, the H_2_O_2_ treatment induced the xylem to consume more NSCs and synthesize more lipids than the other treatments.

### Effect of Chemical Induction on Wood Properties

#### Wood Discoloration

**Figure 5:**
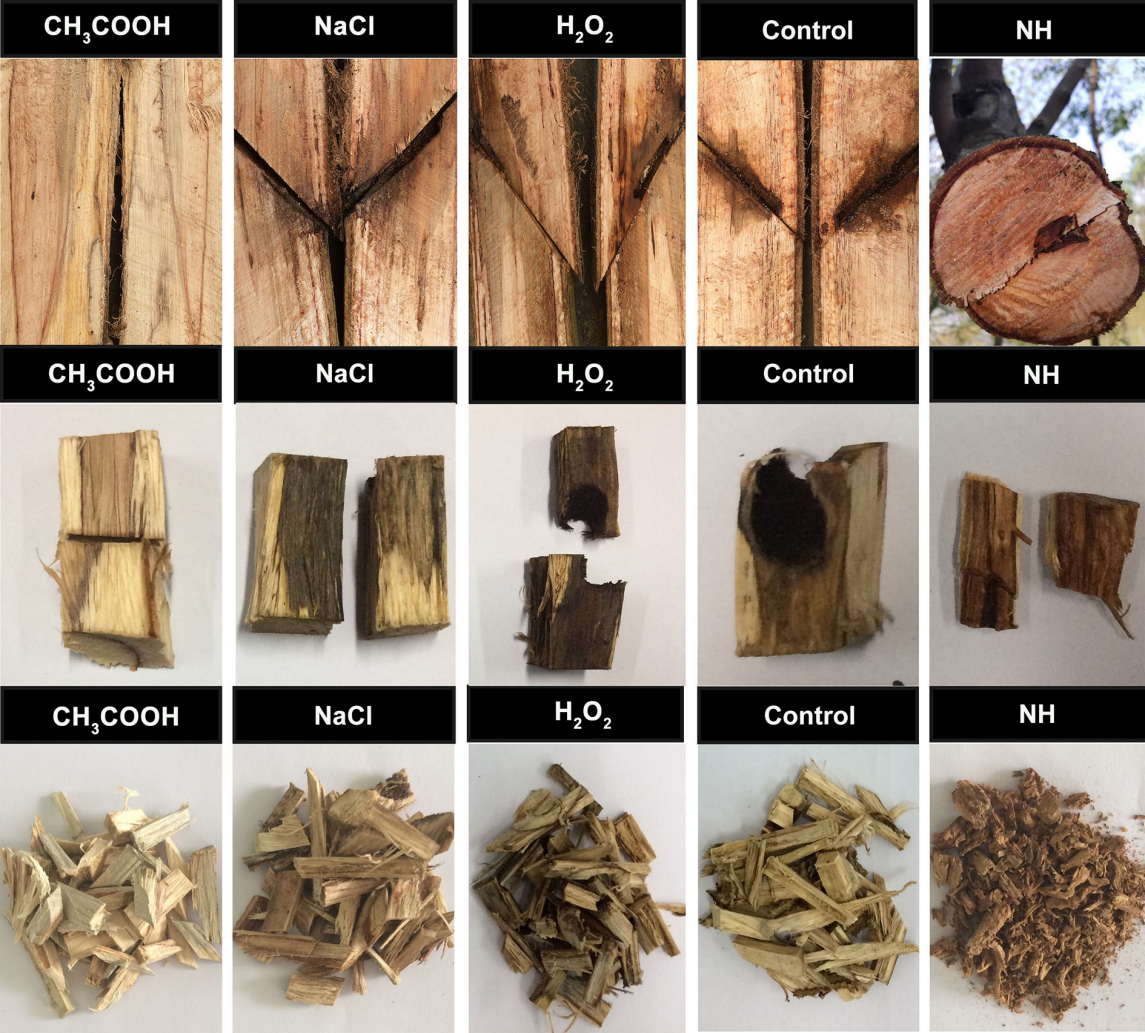
Image description of *Jiang Xiang* of *Dalbergia odorifera*. The images from left to right represent CH_3_COOH, NaCl, H_2_O_2_ and wounding induced *Jiang Xiang* (discolored wood), respectively. NH represents the natural heartwood (wild *Jiang Xiang*), which forms very slowly.

**Figure 6:**
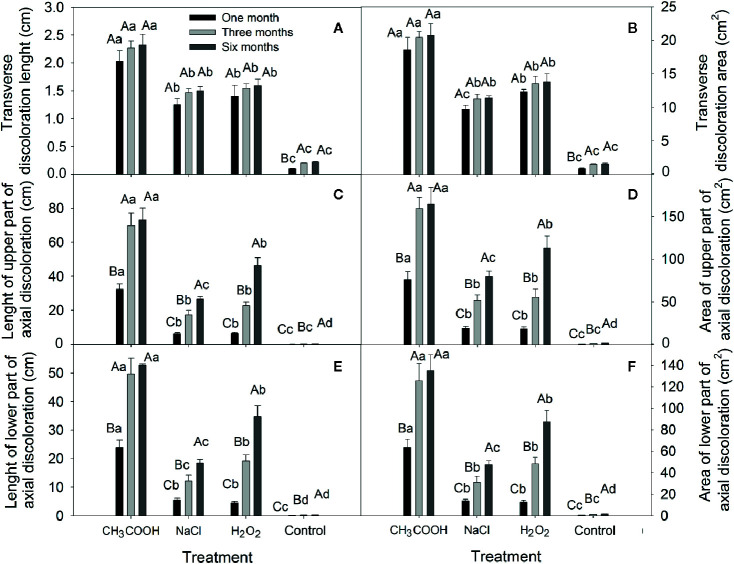
Changes in the transverse and axial discoloration induced by different chemicals. **(A**, **C**, **E)** indicate discoloration length of the transverse, axial-upper, and axial-lower, respectively. **(B**, **D**, **F)** indicate discoloration area of the transverse, axial-upper, and axial-lower, respectively. Similar uppercase letters indicate that the temporal effect is not significantly different. Similar lowercase letters indicate that the treatment effect is not significantly different (*p* > 0.05). Bar represents standard deviation.

In this study, all treatments caused sapwood discoloration (**Figure 5**). The length and area of induced discoloration were significantly different among treatments, and the change in discoloration length was similar to that of the discoloration area (**Figure 6**). The largest discoloration length and area of both transverse and axial discoloration occurred in the CH_3_COOH treatment, followed by the H_2_O_2_ treatment. No significant changes in the transverse discoloration length and area were found with chemical treatments over time. In contrast, the transverse discoloration of the control treatment nearly doubled after three months, and the subsequent increase was small (**Figures 6A, B** **)**.

The length and area of axial discoloration of all treatments increased significantly with the treatment time (**Figures 6C–F**). In the CH_3_COOH treatment, the discoloration length and area after three months were more than double those at one month. The discoloration length and area of the NaCl, H_2_O_2_, and CK plants increased significantly after three and six months. Hence, NaCl, and H_2_O_2_ had a lengthier period of induction effect compared with CH_3_COOH.

#### Relative Moisture Content

**Figure 7:**
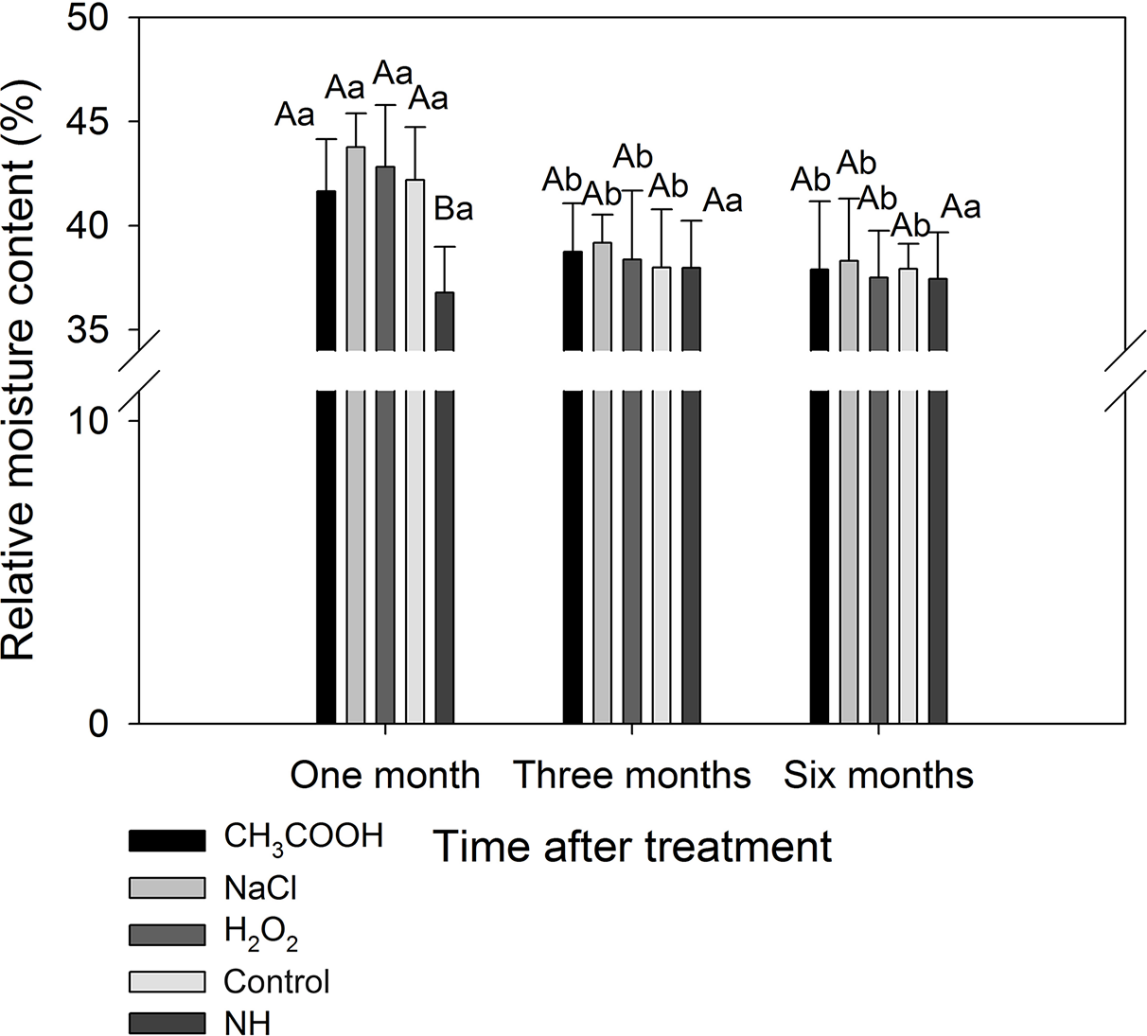
Relative moisture contents of discolored sapwood induced by different chemicals. Similar uppercase letters indicate that the treatment effect is not significantly different. Similar lowercase letters indicate that the temporal effect is not significantly different (*p* > 0.05). Bar represents standard deviation.

After one month of treatment, the relative moisture content of the discolored sapwood induced by each treatment was significantly higher than that of natural heartwood (NH) (*p* < 0.05). The relative moisture content of each treatment was significantly reduced after three months compared to one month of treatment (*p* < 0.05), which were not significantly different from that of NH (**Figure 7**). These results indicated that the water in the sapwood continuously reduced during induced discoloration of the sapwood, and the relative moisture content level approached that of the natural heartwood after three months of treatment.

#### Basic Density

**Figure 8:**
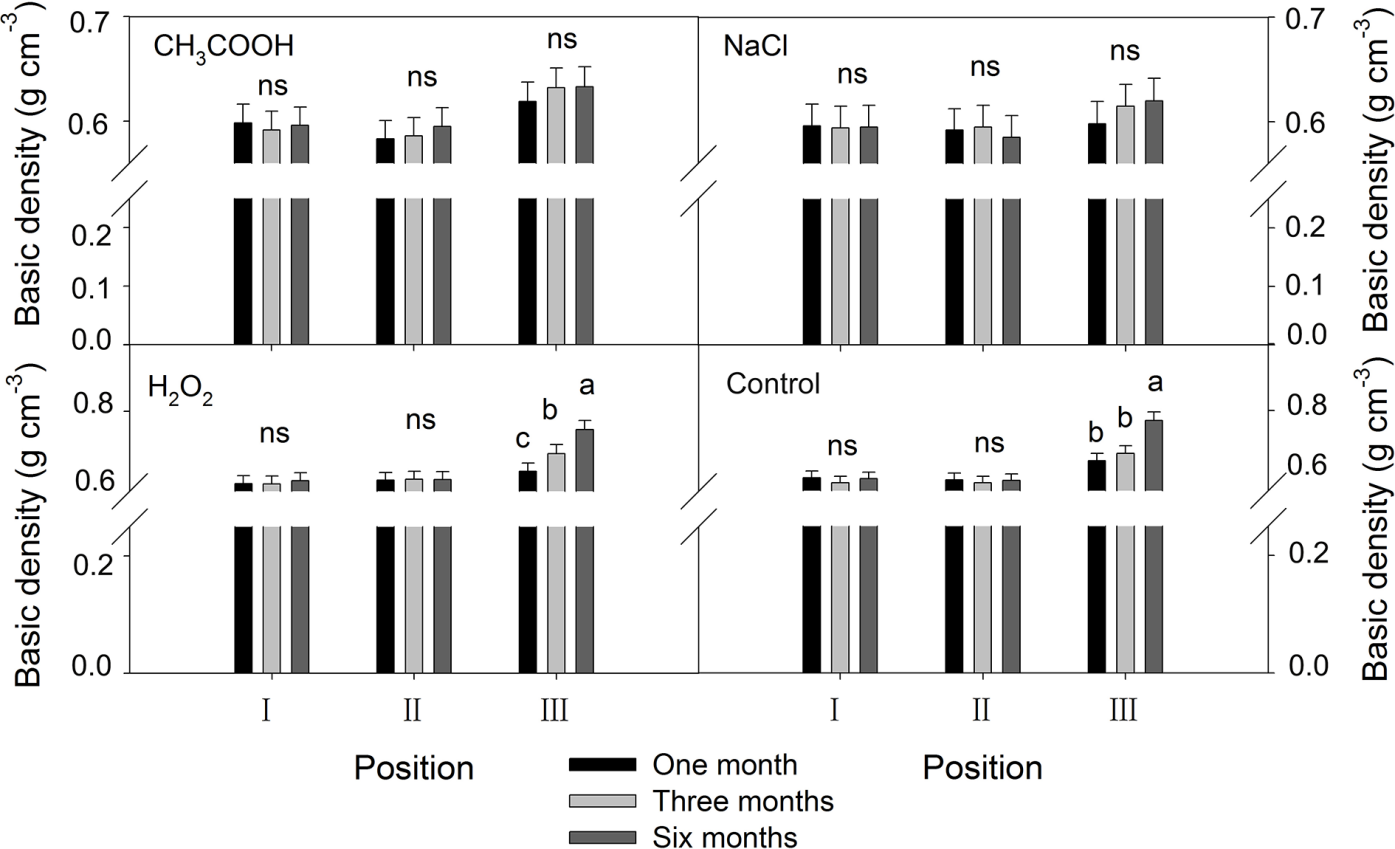
Effects of different chemicals on basic density of parts of the xylem. Similar letters indicate no significant difference at the 0.05 level (*p* > 0.05). “ns” indicates no significant difference at the 0.05 level. Bar represents standard deviation.

As shown in **Figure 8**, no significant changes in the basic density were observed in positions I and II of each treatment over the six months period. In contrast, the basic density of the discolored sapwood (position III) increased significantly with time (*p* < 0.01) in H_2_O_2_ and control treatment but not in CH_3_COOH and NaCl treatments.

**Figure 9:**
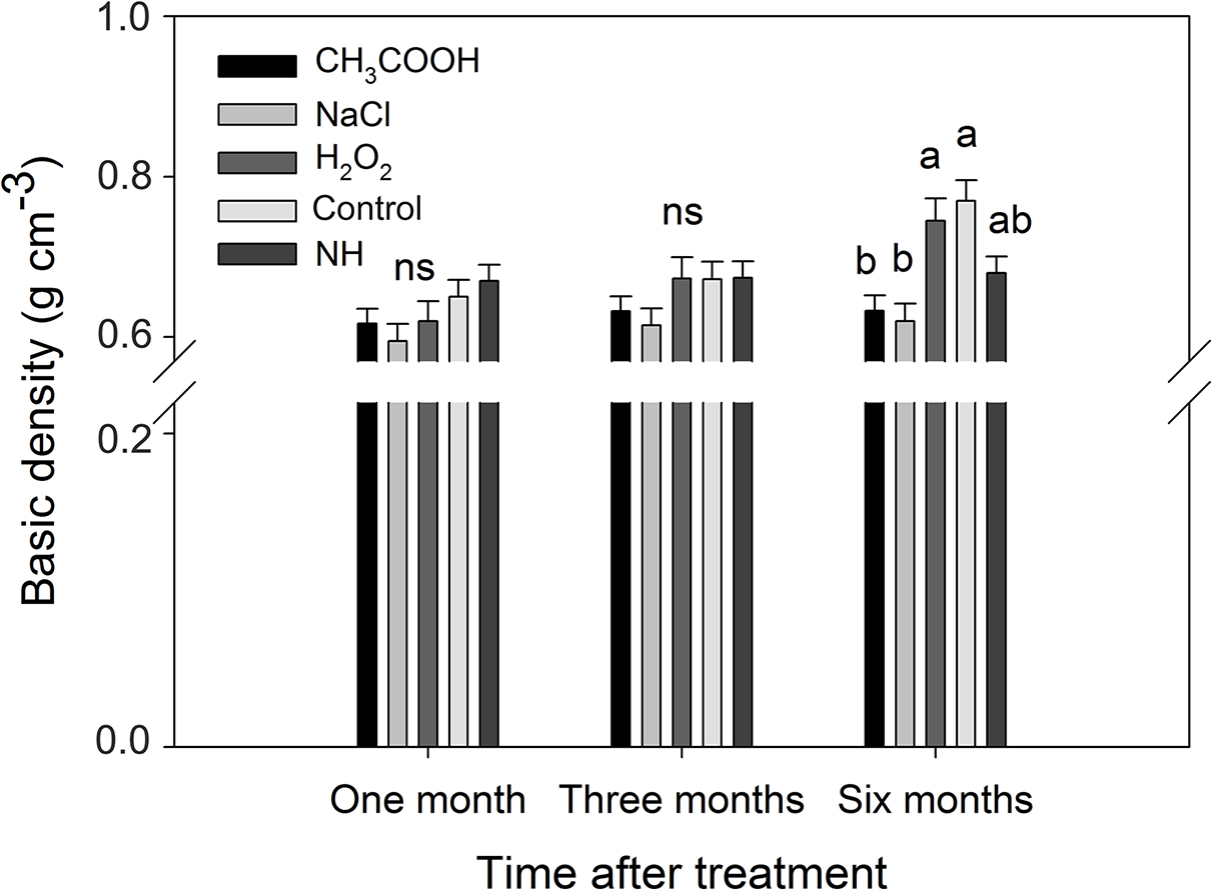
Basic density of discolored sapwood induced by different chemicals. Similar letters and “ns” indicate no significant difference at the 0.05 level (*p* > 0.05). Bar represents standard deviation.

After one and three months of treatment, the basic density of the discolored sapwood in each treatment was not significant different from that of NH. However, the density of the discolored sapwood in the H_2_O_2_ and control treatments was significantly higher than that of NH after six months, while the density of the discolored sapwood in CH_3_COOH and NaCl treated wood remained slightly lower than that of NH (**Figure 9**). These results indicated that the basic density of discolored sapwood gradually increased with time after treatment, but it did not reach the same density level of natural heartwood.

#### Essential Oil Content

**Figure 10:**
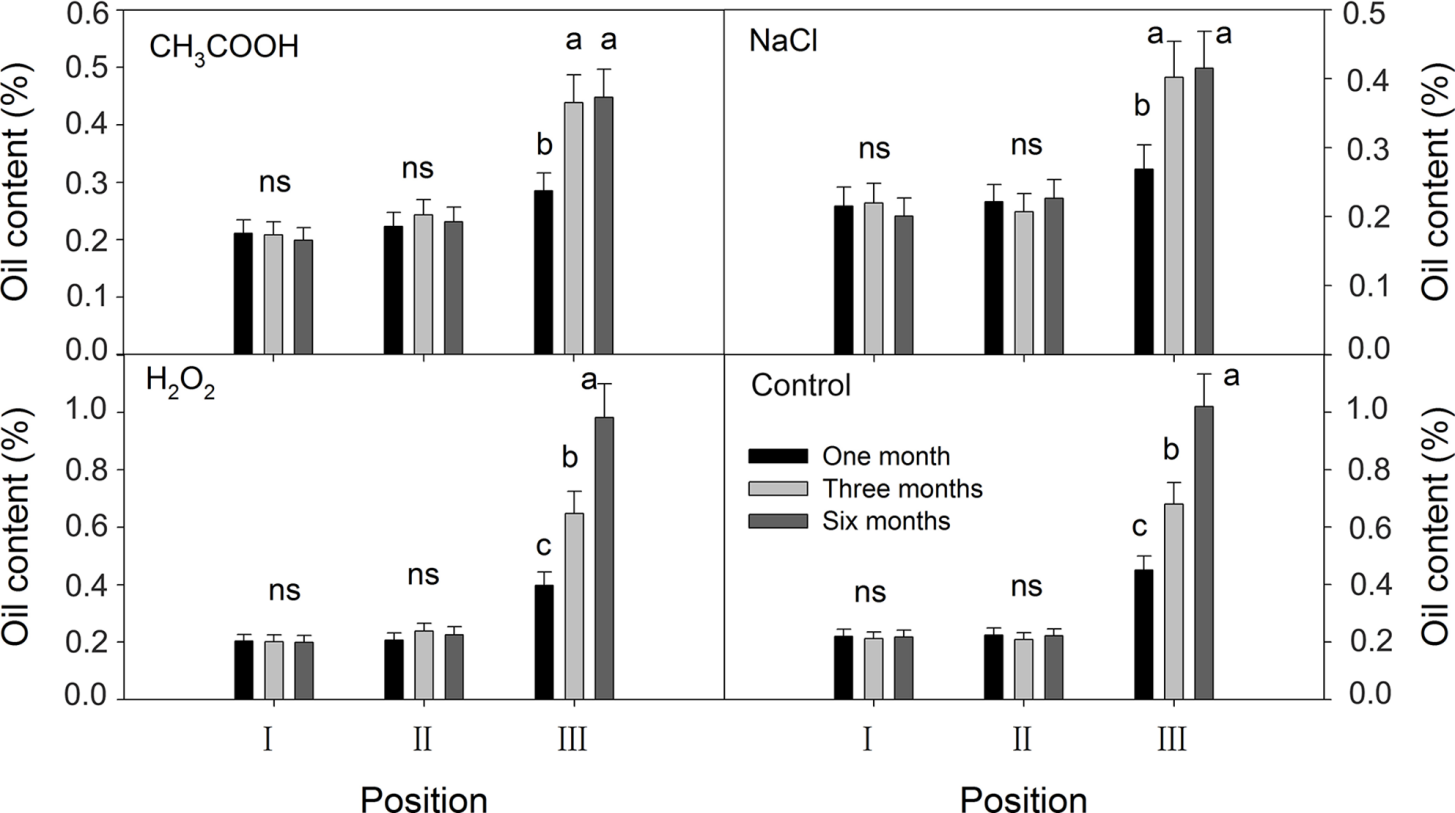
Effects of different chemicals on essential oil contents of parts of the xylem. Similar letters and “ns” indicate no significant difference at the 0.05 level (*p* > 0.05). Bar represents standard deviation.

No significant temporal changes in the oil content were observed in positions I and II of all the treatments; oil content was about 0.20% (**Figure 10**). In position III, the oil contents of the discolored sapwood of all treatments increased significantly from one month to three months after treatment. Further increase in oil content was only significant in the H_2_O_2_ and control treatments.

**Figure 11:**
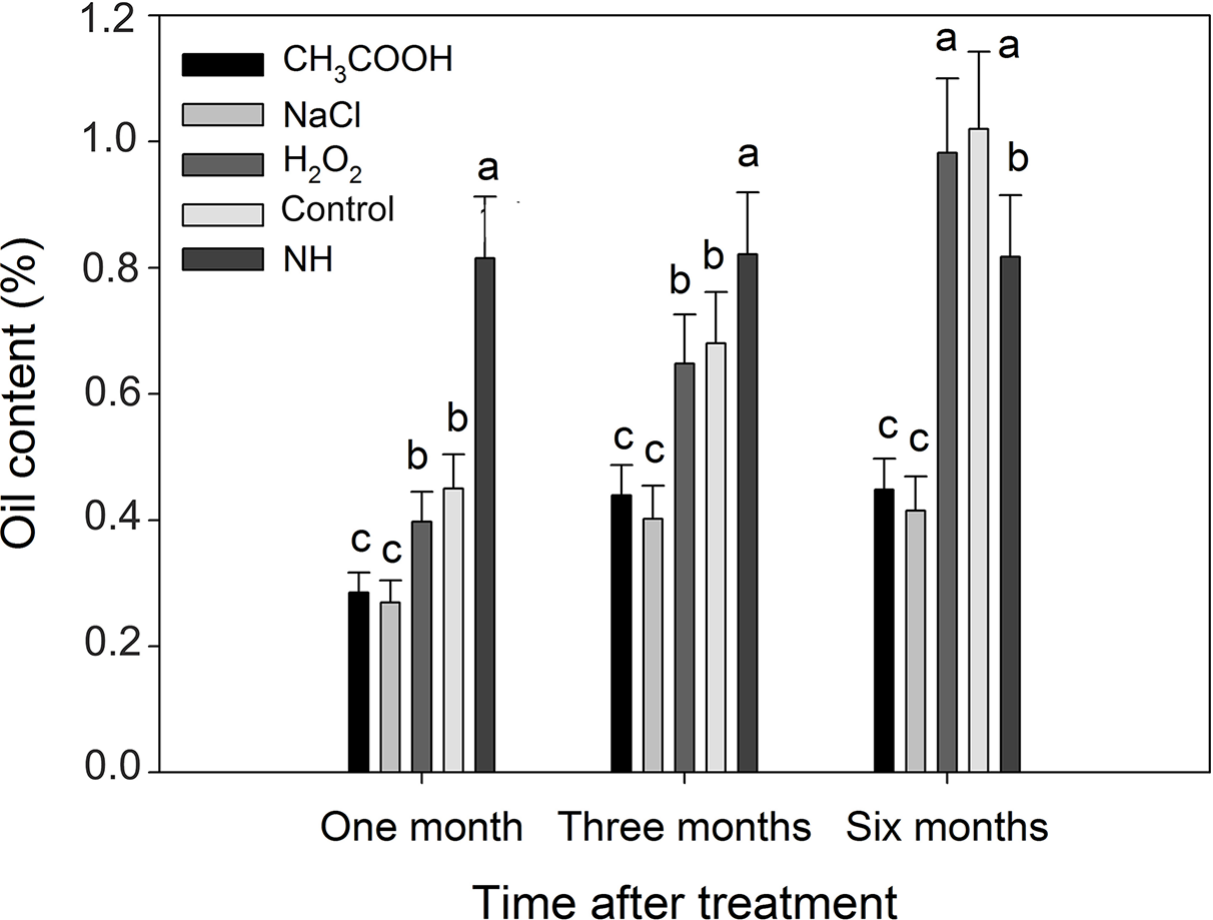
Essential oil contents of discolored sapwood induced by different chemicals. Similar letters indicate no significant difference at the 0.05 level (*p* > 0.05). Bar represents standard deviation.

Compared with natural heartwood, the oil content of induced heartwood was significantly lower after one and three months of treatment (**Figure 11**). After six months, the oil content of the discolored sapwood in the H_2_O_2_ and control treatments, which was 0.98 and 1.02% respectively, was significantly greater (*p* < 0.01) than that of NH (0.82%). However, the oil content of the discolored sapwood induced by the CH_3_COOH and NaCl treatments was still significantly lower than that of NH.

#### Essential Oil Components

Twenty-one essential oil components in the natural heartwood were used as a reference set for comparison with the essential oil components of the natural heartwood. The composition and relative content of essential oil components induced by chemicals increased with the treatment time (**Table 1**). One month after treatment, only 6–8 components of essential oil were detected, and the total relative amount of essential oil components was 38.90–44.65%. After three months, 9–12 components of essential oil were detected, and the total relative amount of essential oil components was 41.27–48.47%. After six months, there were 12 and 14 kinds of essential oil in the CH_3_COOH and NaCl treatments, respectively, and the total relative amount of essential oil components of the NaCl treatment was up to 50.07%. The control treatment, which contained more essential oil components (17), had the total relative amount of the essential oil components at 46.47%.

**Table 1:** Essential oil composition and relative amounts in discolored sapwood induced by different treatments.

RT (min)	Compound name	Molecular formula	Relative amount (%)												
			One month after treatment				Three months after treatment				Six months after treatment				NH
			H_2_O_2_	CC	NaCl	Control	H_2_O_2_	CC	NaCl	Control	H_2_O_2_	CC	NaCl	Control	
11.925	5,9-Undecadien-2-one, 6,10-dimethyl	C_13_H_22_O	–	–	–	–	–	–	–	–	0.05	–	0.08	0.01	0.04
**11.992**	**(E)-.beta.-Farnesene**	**C_15_H_24_**	**–**	**–**	**–**	**–**	**–**	**–**	**–**	**–**	**0.04**	**–**	**0.11**	**0.02**	**0.06**
13.11	6-Octen-1-ol, 7-methyl-3-methylene-	C_10_H_18_O	–	–	–	–	–	–	–	–	0.03	–	–	–	0.05
13.235	Hotrienol	C_10_H_16_O	–	–	–	–	0.06	0.17	–	–	0.06	–	–	0.01	0.12
**13.629**	**trans-Nerolidol**	**C_15_H_26_O**	**36.37**	**38.07**	**36.62**	**36.21**	**38.84**	**39.63**	**38.17**	**38.25**	**39.14**	**35.07**	**37.49**	**39.84**	**39.54**
13.967	7-Oxabicyclo[4.1.0]heptane, 1-methyl-4-(2-methyloxiranyl)-	C_10_H_16_O_2_	–	–	–	–	–	–	–	–	0.09	–	–	–	0.13
14.111	.alpha.-Farnesene	C_15_H_24_	–	–	–	–	–	–	–	0.17	0.07	–	–	0.03	0.08
14.761	2-Cyclohexen-1-ol, 2-methyl-5-(1-methylethenyl)-, cis-	C_10_H_16_O	1.95	2.68	1.40	1.04	2.10	1.71	2.37	1.19	2.02	1.90	2.69	1.94	2.42
**14.901**	**.alpha.-Bisabolol**	**C_15_H_26_O**	**–**	**–**	**–**	**–**	**0.29**	**–**	**0.30**	**–**	**0.12**	**–**	**0.03**	**0.04**	**0.28**
14.987	1,5-Heptadiene, 3,3-dimethyl-, (E)-	C_9_H_16_O	0.82	1.10	1.07	–	0.38	1.08	0.93	0.15	1.48	0.55	0.34	0.24	2.30
15.113	2-Acetyl-1,4,5,6-tetrahydropyridine	C_7_H_11_NO	–	–	–	–	0.35	0.41	1.01	0.28	0.52	0.42	0.27	0.15	0.68
15.195	Bicyclo[3.1.1]heptane, 6,6-dimethyl-3-methylene-	C_10_H_16_	–	–	–	–	0.11	0.29	0.24	0.13	0.17	0.13	0.25	0.10	0.21
15.512	alpha.-Santalol	C_15_H_24_O	–	–	–	–	0.17	0.51	–	–	0.24	0.14	–	0.18	0.26
15.82	2-Isopropenyl-5-methylhex-4-enal	C_10_H_18_O	0.92	0.34	1.03	0.21	0.39	0.65	0.69	–	0.45	0.19	0.90	0.45	0.93
15.936	2,6,10-Dodecatrien-1-ol, 3,7,11-trimethyl-	C_15_H_26_O	–	–	–	–	–	–	–	–	1.46	1.17	2.67	1.20	1.42
16.028	2,7-Octadien-4-ol, 2-methyl-6-methylene-, (S)-	C_10_H_16_O	–	–	–	–	–	–	–	–	0.28	0.15	0.95	0.30	0.23
16.273	2-Cyclohexene-1-carboxaldehyde, 2,6,6-trimethyl-	C_10_H_16_O	1.05	0.87	0.57	0.65	0.93	1.08	2.36	0.56	1.04	0.50	2.80	1.39	2.06
16.557	Citronellol	C_10_H_20_O	0.22	–	–	–	0.13	0.29	0.14	–	0.05	0.10	–	–	0.08
16.779	Butanoic acid, 3-hexenyl ester, (Z)-	C_10_H_18_O_2_	1.41	1.24	1.33	0.65	2.25	1.44	2.17	0.44	1.14	2.45	1.43	0.53	1.92
17.496	3-Decyn-1-ol	C_10_H_18_O	–	–	–	–	–	–	–	–	0.12	–	–	–	0.16
19.74	Nerolidol	C_15_H_26_O	0.15	0.35	0.17	0.14	–	–	0.09	0.10	0.02	–	0.06	0.04	0.03
**Compound number**			8	7	7	6	12	11	11	9	21	12	14	17	21
**Total(%)**			42.89	44.65	42.19	38.90	46.00	47.26	48.47	41.27	48.59	42.77	50.07	46.47	53.00

”–” means not detected. RT, Retention time; CC, CH_3_COOH; NH, natural heartwood. Bolded texts indicate the signature ingredient in the essential oils of high-quality Jiang Xiang.

All 21 essential oil components in the natural heartwood were detected in the H_2_O_2_ treatment with the relative amount reaching 48.59%. Moreover, the relative amount of trans-Nerolidol (39.14%), (E)-beta-Farnesene (0.04%), and alpha-Bisabolol (0.12%) in the H_2_O_2_-induced essential oil met the standards for high-quality *Jiang Xiang*, *i.e.* 25–60%, 0–3%, and 0.1–6.0%, respectively. The relative amount of alpha-Bisabolol in the other treatments was considerably less at 0.03 and 0.04%. Therefore, a greater diversity of heartwood oil components was induced by the H_2_O_2_ treatment than by the CH_3_COOH and NaCl treatments. H_2_O_2_ was the only inducer of essential oil components that reached the level of NH and met the standards of high-quality *Jiang Xiang*.

## Discussion

### Physiological Parameters of Discolored Sapwood Gradually Evolve to Resemble Those of Natural Heartwood During Chemical Induction

After chemical treatment, an overall trend of a decrease in non-structural carbohydrate content and an increase in lipid content was observed in xylem. Similar results were observed in a study of growth regulator-induced sapwood discoloration of *Quercus serrata* (Moungsrimuangdee et al., 2011) and consistent with the chemical changes during the formation of natural heartwood (Magel et al., 1994). The depletion of storage starch was thought to be associated with parenchyma cells’ death, and this relationship controlled the formation of heartwood (Islam et al., 2012). When the xylem was exposed to these chemicals, large amounts of phytoalexins (heartwood substances) were synthesized in the parenchyma cells and transported into the infected vessels to inhibit further infection. As precursors, the NSCs supplied a large quantity of carbon skeletons for the synthesis of heartwood substances (Magel et al., 1994). In addition, these chemicals may affect the storage functions of NSCs by altering the pH of the xylem (Pagliarani et al., 2019). After one month of treatment, the levels of NSCs in positions II and III decreased significantly, while those in position I did not decline until after six months when the NSCs in position III were almost exhausted. These results indicated that when the NSCs in position III were consumed to a certain extent, the NSCs in positions II and I were successively consumed. Since no lipid accumulated in position I, it was speculated that the NSCs in position I might be transferred to position II or III in wood ray parenchyma. Some studies have suggested that NSCs could be transported in both directions in wood ray parenchyma (Furze et al., 2018). The NSCs transported outward could maintain the “Leakage-retrieval Mechanism” of phloem (De Schepper et al., 2013), and the NSCs transported inward might be used as substrate for secondary metabolism in xylem, such as for the synthesis of heartwood substances (Magel et al., 1991). In addition, stachyose, galactose, and arabinose were exhausted first, which might be related to their characteristics and functions. These oligosaccharides or monosaccharides are small storage materials and are the most easily utilized carbohydrates in trees (Saranpää and Höll, 1989). Interestingly, after six months of treatment, the arabinose and galactose contents increased in position III of each treatment. The arabinose and galactose produced in position III might be derived from partial hydrolysis of hemicellulose in the cell wall during the formation of heartwood (Saranpää and Höll, 1989; Fischer and Höll, 1992). Galactose has been reported to stimulate the production of ethylene in tomato fruits (Kim et al., 1987), and ethylene was thought to be closely associated with heartwood formation (Hillis, 1987; Cui et al., 2019). Therefore, NSCs can directly provide raw materials for the synthesis of heartwood substances and also indirectly regulate physiological processes related to heartwood formation. In general, as observed in the formation of natural heartwood, in the process of chemical induction, NSCs were continuously consumed, and heartwood substances were gradually synthesized in xylem, while hemicellulose in the xylem cell wall might have been partially hydrolyzed. However, the metabolic characteristics and physiological functions of specific carbohydrates involved in the synthesis of heartwood substances are little known and deserve further study.

In terms of wood properties, the relative moisture content of the discolored sapwood continued to decrease, while the density and oil content increased gradually during the induction process. Dehydration of sapwood was an important stage in the formation of heartwood (Nakada, 2006; Kuroda et al., 2009; Nakada and Fukatsu, 2012), which accelerated the decline of sapwood cells (Spicer and Holbrook, 2005), stimulated ethylene synthesis (Shigo and Hillis, 1973), or altered xylem water distribution (Watanabe et al., 2012), and finally induced heartwood formation (Nakada and Fukatsu, 2012). In return, the deposition of heartwood substances blocked the vessels, which further reduced the moisture content (Taylor et al., 2002; Déjardin et al., 2010). Furthermore, the deposition increased the xylem density and oil content. These results are consistent with the density and oil content of heartwood being greater than that of sapwood (Taylor et al., 2002; Searle and Owen, 2005). In addition, GC–MS analysis also showed that the essential oil components of the discolored sapwood became progressively more similar to those of natural heartwood.

Comprehensive analysis of NSCs, lipids, and wood properties showed that, as observed in the formation process of natural heartwood, chemical-induced sapwood discoloration was accompanied by sapwood dehydration, NSC consumption and synthesis of heartwood substances. These substances filled and blocked the lumen of the vessels, which further reduced the moisture content of the discolored sapwood. As the heartwood substances accumulated, the density, essential oil content, and components of the discolored sapwood increased gradually, and thereby *Jiang Xiang* was finally formed.

### H2O2 Is a Promising Candidate for Artificially Induced Jiang Xiang in *D. odorifera*

This study showed that the injection of an acid solution into the trunk markedly inhibited growth of the tree, while the injection of distilled water, NaCl solution, and H_2_O_2_ solution into the trunk had little or no effect. Wang (2016) found that acid solution destroyed the tissue cells of the xylem, and thus, it inhibited the growth and even caused tree death. The comprehensive analysis of the transverse and axial discoloration ranges indicated that the range of discoloration induced by CH_3_COOH was the largest, followed by the H_2_O_2_ treatment. In terms of the persistence of the induction effect, some differences were found between the transverse and axial directions. The effects of transverse discoloration induced by all treatments were only maintained for one month; however, in the axial direction, the induction effect of the CH_3_COOH treatment lasted for three months, and the H_2_O_2_ and NaCl treatments still had induction effects after six months. Moreover, the H_2_O_2_ treatment induced xylem to consume most of the NSCs and synthesize most of the lipids, and its essential oil content and basic density reached the levels of natural heartwood. By contrast, although the density of the discolored sapwood induced by the CH_3_COOH and NaCl treatments reached the level of natural heartwood, their essential oil contents were still much lower than those of natural heartwood. In addition, the essential oil components of H_2_O_2_-induced discolored sapwood were closest to those of natural heartwood. Therefore, H_2_O_2_-induced *Jiang Xiang* is closest to natural heartwood, while the CH_3_COOH and NaCl treatments have poor induction effects. Moreover, the relative amount of trans-Nerolidol (39.14%), (E)-beta-Farnesene (0.04%), and alpha-Bisabolol (0.12%) in the H_2_O_2_-induced essential oil met the standards for high-quality *Jiang Xiang* which are 25–60%, 0–3%, and 0.1–6.0%, respectively. Thus, H_2_O_2_ is a promising inducer for *Jiang Xiang* production in *D. odorifera*. The superior effect of H_2_O_2_ treatment may be related to its involvement in the wound signal of *D. odorifera* (Cui et al., 2019), which in turn illustrates the important role of H_2_O_2_ in wound-induced *Jiang Xiang* formation. This study addressed some of the key factors related to heartwood formation and the results indicate the potential of for H_2_O_2_ to induce high-quality *Jiang Xiang* production. Further study is warranted to explore the molecular mechanism of H_2_O_2_ regulation on *Jiang Xiang* formation in *D. odorifera*. Furthermore, the chemicals used in this study are only in certain doses, and a wider range of concentrations needs to be examined. At present, the production of *Jiang Xiang* is extremely limited. The induction of H_2_O_2_ will greatly increase the yield and will have commercial potential if the techniques can be further improved and widely implemented.

## Conclusions

All chemical inducers in this study are effective in inducing discoloration of sapwood. In this process, physiological parameters of discolored sapwood gradually evolved to resemble those of natural heartwood. Comparative analysis indicated hydrogen peroxide (H_2_O_2_)-induced *Jiang Xiang* was closest to natural heartwood and met the standards for high-quality *Jiang Xiang*, while the induction effects of CH_3_COOH and NaCl were unsatisfactory. Thus, this study supports the hypothesis that H_2_O_2_ has the potential to induce formation of *Jiang Xiang* in *D. odorifera*.

## Data Availability Statement

The datasets generated for this study are available on request to the corresponding author.

## Author Contributions

ZC developed the study design and wrote the manuscript. XL and ZY helped conduct data collection and analysis. DX provided expert knowledge in the writing and revision of the manuscript.

## Funding

This work was financially supported by the Fundamental Research Funds for the Central Non-profit Research Institution of the Chinese Academy of Forestry (Grant No. CAFYBB2017ZX001-4) and the National Key Research and Development Program of China (2016YFD0600601).

## Conflict of Interest

The authors declare that the research was conducted in the absence of any commercial or financial relationships that could be construed as a potential conflict of interest.
